# IgG4-related disease can present as recurrent spontaneous hemothorax: a case report

**DOI:** 10.1186/s12890-019-0785-y

**Published:** 2019-02-01

**Authors:** Junping Fan, Ruie Feng, Xiaomeng Hou, Ji Li, Xuefeng Sun, Jinglan Wang, Juhong Shi, Mengzhao Wang, Yan Xu

**Affiliations:** 1 Department of Respiratory Medicine, Peking Union Medical College Hospital, Chinese Academy of Medical Sciences & Peking Union Medical College, Beijing, China; 2 Department of Pathology, Peking Union Medical College Hospital, Chinese Academy of Medical Sciences & Peking Union Medical College, Beijing, China

**Keywords:** Immunoglobulin G4-related disease, Spontaneous hemothorax, Pleura involvement

## Abstract

**Background:**

Immunoglobulin G4-related disease (IgG4-RD) encompasses a group of immune-mediated disorders that are gaining increasing recognition. Pulmonary presentations are common, with four types of patterns been described on radiography, including solid nodular, bronchovascular, ground glass opacities, and alveolar interstitial. Pleural thickening and pleural effusion have also been reported. However, there have been no reports of IgG4-RD that presents as spontaneous hemothorax.

**Case presentation:**

A 61-year-old Chinese woman experienced recurrent right-sided chest pain and transient syncope. A significant decrease in her hemoglobin level and thick bloody pleural fluid demonstrated spontaneous hemothorax. The elevated serum IgG4 and histopathological analysis of the right pleura and intercostal lymph node specimens all supported the diagnosis of IgG4-RD in this patient. Further diagnostic evaluation did not reveal other causes for spontaneous hemothorax. She received steroids and no recurrent bleeding event occurred during a follow-up period of more than 1 year.

**Conclusion:**

Recurrent spontaneous hemothorax can be a rare manifestation of IgG4-RD, with pleural involvement as the most probable mechanism.

## Background

Immunoglobulin G4-related disease (IgG4-RD) encompasses a group of immune-mediated disorders that are gaining increasing recognition. It can involve one or multiple organs, including the salivary glands, orbital soft tissues, mediastinum, lungs, pleura, lymph nodes, large arteries, pancreas, eyes, thyroid etc. [1] Elevated serum IgG4 level could be a useful indicator for further diagnostic studies given the context of possible organ involvement. Nevertheless, definite diagnosis depends on biopsy and histopathologic findings. Common pathological features include a dense lymphoplasmacytic infiltrate rich in IgG4-positive plasma cells, and varying degrees of storiform fibrosis, and obliterative phlebitis. Multiple reports [2, 3] have demonstrated IgG4-related pulmonary disease, which may be asymptomatic, or present with cough, dyspnea, hemoptysis, chest pain, and constitutional symptoms such as fever. Radiological patterns of IgG4-related pulmonary disease were described as solid nodular, bronchovascular, ground glass opacities and alveolar interstitial. Pleural thickening and pleural effusion were also reported but are generally rare. Herein, we present a rare case of IgG4-related disease that caused spontaneous hemothorax.

### Case presentation

A 61-year-old Chinese woman was admitted to the Department of Respiratory Medicine, Peking Union Medical College (PUMC) Hospital with recurrent chest pain and transient syncope. Nine months before her admission, she had developed sudden burning chest and back pain on the right side, along with transient syncope, sweating, fatigue, and dizziness. She was admitted to a local hospital, where hypotension (85/50 mmHg) was detected. Laboratory studies showed a significant decrease in her hemoglobin level (93 g/L to 50 g/L). Thoracic ultrasound revealed right-sided pleural effusion with septa while chest computed tomography (CT) also showed right-sided encapsulated pleural effusion. Her symptoms improved after supportive treatment, and the effusion was seen to be gradually absorbed on serial follow-up CT scans. However, the cause of the chest pain, syncope, and anemia remained unclear.

Furthermore, in the 12 years previous to that visit, the patient had developed recurrent periorbital nodules and a submaxillary mass, and had undergone multiple surgeries in other hospitals. Biopsies of these lesions revealed inflammatory pseudotumor, dacryoadenitis (periobital), and reactive lymph node (submaxillary) hyperplasia. She exhibited an improvement with steroid treatment, but we were unable to obtain detailed records of her treatment regime. The patient had no history of hypertension, diabetes, hepatitis, or tuberculosis. She took no medication on a regular basis and had never smoked or used illicit drugs.

**Fig. 1:**
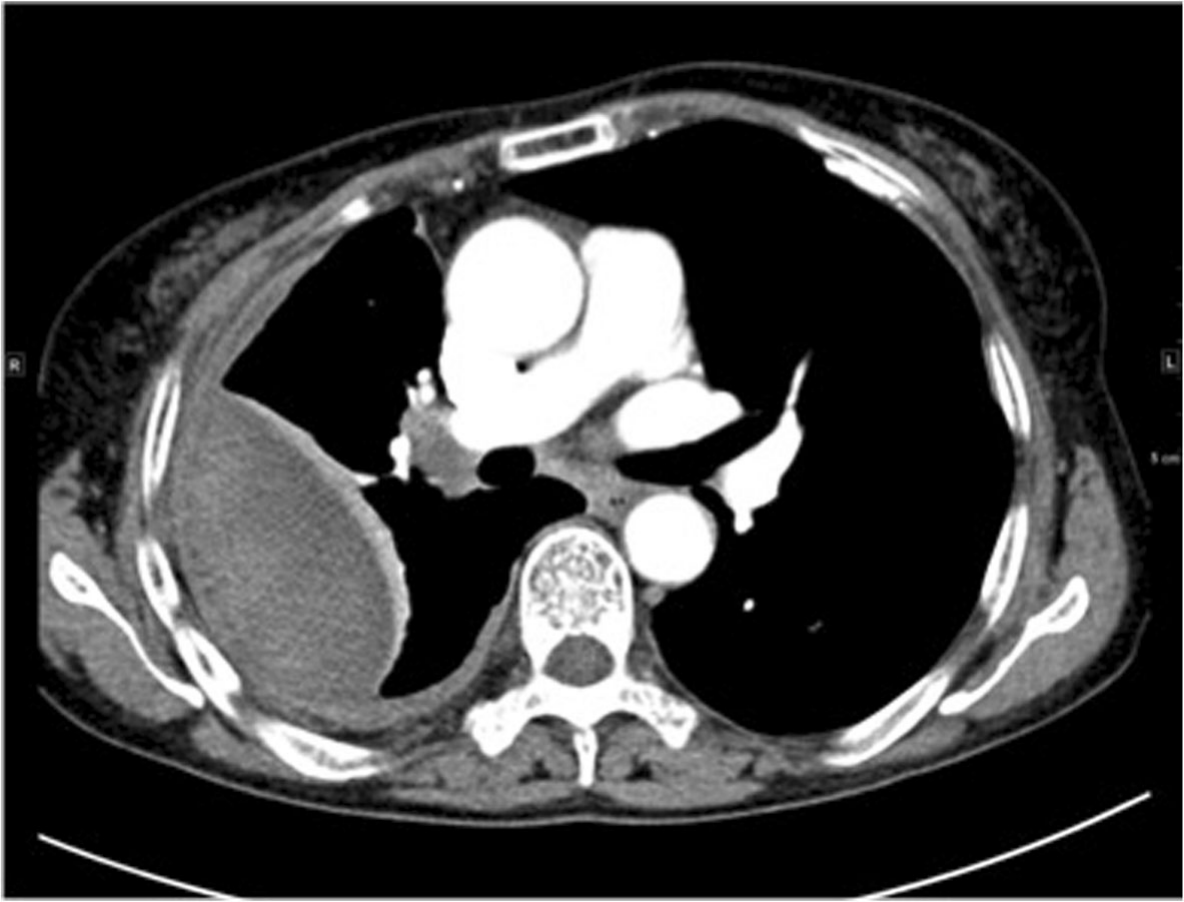
Chest CTA showed encapsulated pleural effusion with atelectasis on the right side, multiple enlarged lymph nodes could be seen in right intercostal, right hilar and mediastinal area

Earlier on the day of her admission to our hospital, the patient experienced another similar episode of chest pain along with sweating and transient syncope. She was sent to the ER of a local hospital and given fluid resuscitation, then transferred to our hospital. On examination, her blood pressure was 110/61 mmHg, and her electrocardiogram was normal. Her hemoglobin level was found to have decreased from 121 g/L to 71 g/L. Physical examination revealed anemia, scars and yellowish nodules on the eyelids, one hemorrhagic vesicle on the buccal mucosa, and skin bruises. Breath sounds over the right lower lung were diminished. Thoracentesis revealed thick bloody fluid with clots, and pleural fluid analysis showed an increase in the total cell number (1.763 × 10^12^/L), white blood cell count (5.74 × 10^9^/L), total protein (TP) level (154 g/L), and lactate dehydrogenase (LDH) level (13,995 U/L). Other laboratory tests showed a significant increase in the C-reactive protein (CRP) level (31.99 mg/L), erythrocyte sedimentation rate (ESR; 118 mm/h), and bleeding time (BT; 21 min). Chest CT showed encapsulated pleural effusion with atelectasis on the right side, and multiple enlarged lymph nodes in the right intercostal, right hilar, and mediastinal area (Fig. 1). CT angiography (CTA) showed that the right intercostal arteries were slightly dilated, but did not reveal a potential cause of bleeding such as arteriovenous malformation (AVM) or aneurysm. PET-CT showed thickened right-sided pleura with diffuse SUV elevation. Exploratory surgery was performed, and the specimens that were obtained from the right pleura and intercostal lymph node showed no sign of neoplasia.

**Fig. 2:**
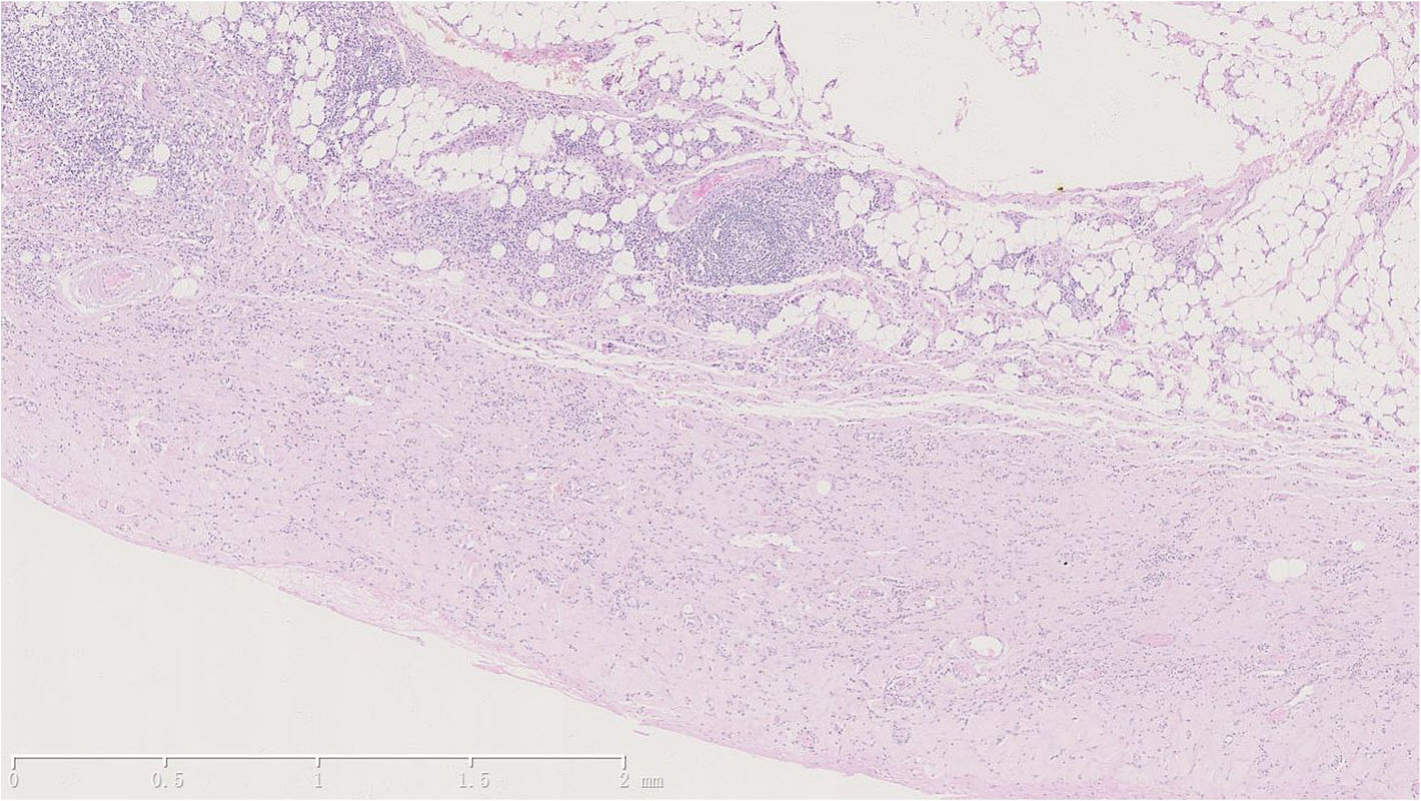
Histopathological features of the right parietal pleura. Dense fibrosis of the pleura extends into the fatty tissue, among lymphoplasmacytic infiltrates a lymphoid follicle can be seen. Small blood vessels are proliferated with lumen dilation and blood congestion. HE × 40

**Fig. 3:**
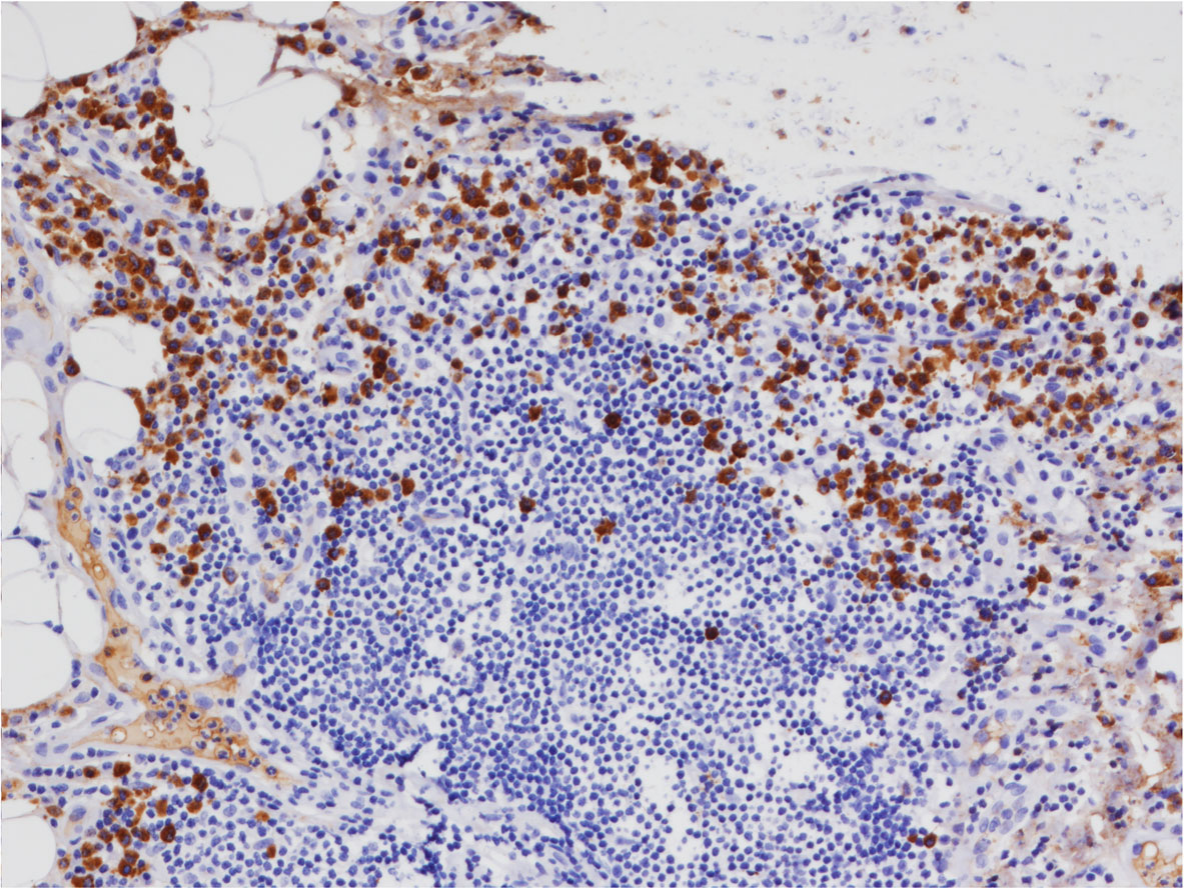
Immunohistochemical staining for IgG4 reveals IgG4-positive plasma cells > 50/HPF. × 200

Based on the patient’s past medical history, IgG4-RD was suspected, and the serum IgG4 test showed a significant increase in IgG4 level (17,400 mg/L). The histopathological analysis, conducted by a specialized pathologist, of the right parietal pleura found dense fibrosis of the pleura, lymphoplasmacytic infiltrates, and proliferated small blood vessels with lumen dilation and congestion (Fig. 2). Immunohistochemical staining showed that all the sampled tissues (right pleura and intercostal lymph node specimens, together with previously biopsied periorbital mass specimens) were infiltrated by massive lymphocytes and plasma cells, with more than 50 IgG4-positive cells per HPF (high-power field), and an IgG4-positive cells to IgG-positive cells ratio of more than 40% (Fig. 3).

This patient was diagnosed with IgG4-RD after discussions with multidisciplinary experts. She was treated with prednisone 60 mg per day for 4 weeks, which was then tapered by 5 mg every week to 30 mg per day, and tapered more slowly thereafter. The patient adhered well to the therapy and no severe side effects occurred. At follow-up visits, regression of the periorbital nodules, and mediastinal and intercostal lymph nodes was confirmed. CRP level, ESR, BT, and the IgG4 level returned to normal. More than 1 year later, there had been no recurrent bleeding event, and chest CT performed at the last examination revealed no obvious abnormalities such as pleural effusion, nodules, solid infiltration or lymphadenopathy.

## Discussion and conclusions

Multiple organs can be involved in IgG4-RD, presenting with various clinical symptoms. Among various types of pulmonary presentation, pleural involvement has been less common. In the present case, the two episodes of chest pain and transient syncope were considered to be associated with spontaneous hemothorax, which can explain the encapsulated pleural effusion, dramatic decrease of hemoglobin level, and hypotension. This was confirmed by thoracentesis. Because no provoking factors such as trauma are recorded, the bleeding events were considered to be spontaneous. Spontaneous hemothorax is an uncommon condition with different etiologies, including vascular ruptures, arteriovenous malformation, malignancies, anticoagulation medications, pulmonary infarctions, and hematological abnormalities such as hemophilia [4]. However, after careful history taking and diagnostic evaluations (blood test, CTA, PET-CT, etc.), no other causes for spontaneous hemothorax were revealed.

Additionally, the skin and soft tissue presentation, elevated IgG4 level, the pathological evaluation of pleura and intercostal lymph node, and the treatment outcome all support the diagnosis of IgG4-RD in this patient. Whether IgG4-RD caused recurrent thoracic bleeding is questionable at first glance. However, we postulated that IgG4-RD was the cause of spontaneous hemothorax in this patient because of the following reasons. First, reassessing the patient’s medical history in light of the current diagnosis reveals that she had developed symptoms and signs of IgG4-RD years before the onset of hemothorax. Second, careful diagnostic evaluations revealed no other causes for spontaneous hemothorax, such as hemophilia, arteriovenous malformations, or malignancies. Third, after a careful review of the literature and discussion with multidisciplinary experts, we were unable to postulate an alternative condition that causes hemothorax and IgG4-RD at the same time. Fourth, the structures adjacent to the hematoma (pleura) were explicitly affected by IgG4-RD, and evidence of small vessel involvement was found in the parietal pleura specimen. Finally, our speculation was confirmed by the normal BT and lack of recurrent bleeding event for more than 1 year after steroid treatment.

The exact mechanism underlying the development of spontaneous hemothorax in association with IgG4-RD remains unclear. Li et al. [5] reported that IgG4-RD can cause acquired hemophilia with a prolonged activated partial thromboplastin time (APTT) and suppression of Factor VIII. Although the present patient exhibited mucosal and skin hemorrhages and a prolonged BT, her APTT, prothrombin time (PT), and platelet counts were normal. These findings suggest that the bleeding was largely associated with vessels. Large vessels, including the aorta, have been demonstrated as target organs of IgG4-RD. A cohort study [6] showed that 36 (22.5%) of 160 patients with IgG4-RD had large-vessel involvement. Thirteen patients (36%) had primary IgG4-related vasculitis and aortitis with aneurysm formation as the most common manifestation. Three aneurysms were complicated by aortic dissection or perforations that caused bleeding events. However, CTA showed no aneurysm in our patient. Pleural involvement was evident, in addition to lymphoplasmacytic infiltration, proliferation and dilation of pleural vessels inside the pleura were obvious. In fact, the patient’s clinical presentation implied that smaller arterial branches, probably those located within the pleura, were more likely to be the culprit vessels, considering that the hemorrhage was massive and quick but ceased spontaneously.

There are multiple case reports [7, 8] of IgG4-RD presenting with pleural effusion or pleural lesions. However, to our knowledge, there is no report of IgG4-RD causing thoracic bleeding. In conclusion, we reported a very rare case of IgG4-RD, adding recurrent spontaneous hemothorax as a novel manifestation of this uncommon disease. The most probable mechanism might be pleural involvement by IgG4-RD. Because hemothorax can be lethal, this peculiar presentation in IgG4-RD is worthy of attention.

## Abbreviations

APTT: Activated partial thromboplastin time
BT: Bleeding time
CRP: C-reactive protein
CTA: Computed tomography angiography
ESR: Erythrocyte sedimentation rate
IgG4-RD: Immunoglobulin G4 related disease
LDH: Lactate dehydrogenase
PT: Prothrombin time
TP: Total protein
